# Urine Gram Stain as a New Marker in Predicting Postoperative Fever in Patients Undergoing Endourological Surgeries for Stone Disease: A Prospective Study

**DOI:** 10.7759/cureus.92520

**Published:** 2025-09-17

**Authors:** Vinay S Kundargi, Dhruva HM, Siddanagouda B Patil, Santosh Patil

**Affiliations:** 1 Urology, Shri BM Patil Medical College, Hospital and Research Centre, BLDE (Deemed to be University), Vijayapura, IND

**Keywords:** endourological surgery, percutaneous nephrolithotomy (pcnl), postoperative fever, retrograde intrarenal surgery (rirs), stone disease, ureterorenoscopy (urs), urine gram stain

## Abstract

Introduction: Endourological procedures, including ureterorenoscopy (URS), percutaneous nephrolithotomy (PCNL), and retrograde intrarenal surgery (RIRS), are the standard of care for managing urolithiasis but remain associated with postoperative fever (POF). POF is often linked to preoperative bacteriuria or infected stones and can precede urosepsis. While urine culture is the gold standard for detecting urinary tract infections, its 72-hour delay limits perioperative decision-making. Urine Gram stain (UGS) is a rapid and inexpensive diagnostic tool capable of detecting bacteriuria within minutes. This study aimed to evaluate the predictive value of preoperative UGS for POF in patients undergoing endourological surgeries for stone disease.

Materials and methods: This prospective study enrolled 150 adult patients undergoing URS, PCNL or RIRS for urolithiasis between January 2024 and July 2025. Patients with ongoing sepsis, recent antibiotic use within the preceding 14 days, positive preoperative urine culture, postoperative fever due to other identifiable causes, or incomplete clinical/microbiological data were excluded. Preoperative midstream urine samples were analysed for Gram stain and culture. POF was defined as a temperature ≥38 °C within 72 hours postoperatively, with follow-up extending to capture fevers occurring after discharge during this period. Statistical analysis included chi-square, t-tests, logistic regression, and receiver operating characteristic (ROC) curve analysis to assess the predictive performance of UGS.

Results: POF occurred in 24 patients (16.0%). Positive UGS was observed in 19/24 (79.2%) POF cases versus 21/126 (16.7%) non-POF cases (OR 11.8, 95% CI 4.1-34.2, p<0.0001). Positive urine culture was seen in 16/24 (66.7%) versus 18/126 (14.3%) (OR 5.9, 95% CI 2.1-16.7, p=0.001). UGS demonstrated 79.2% sensitivity, 82.5% specificity, 47.5% positive predictive value, and 94.5% negative predictive value for predicting POF (AUC=0.83, p<0.001). Multivariate analysis confirmed positive UGS (OR 11.8, 95% CI 4.1-34.2) and positive culture (OR 5.9, 95% CI 2.1-16.7) as independent predictors of POF.

Conclusion: Preoperative UGS is a rapid, cost-effective, and reliable predictor of POF in endourological stone surgeries. Its integration into preoperative protocols can enhance early risk stratification, enable timely antibiotic optimisation, and potentially reduce postoperative infectious morbidity.

## Introduction

Urolithiasis is a common urological disorder affecting about one in 11 people worldwide, with a rising incidence reported across 204 countries [1]. It frequently necessitates surgical intervention when conservative management fails or when complications such as obstruction, infection, or recurrent pain arise [1]. With advancements in minimally invasive techniques, endourological procedures such as ureterorenoscopy (URS) and percutaneous nephrolithotomy (PCNL) have emerged as the mainstay treatments for renal and ureteral calculi, offering high stone clearance rates with reduced morbidity compared to open surgery [2]. Despite these advantages, postoperative complications remain clinically significant, with postoperative fever (POF) being one of the most common concerns. The incidence of POF in endourological surgeries has been reported to range from 2% to 20%, depending on patient risk factors, stone burden, and procedural complexity [3].

POF is a critical early warning sign for postoperative urinary tract infection (UTI) and may precede urosepsis, a potentially life-threatening complication. Identifying patients at high risk for infectious complications before or during surgery is therefore crucial for optimising perioperative management [4]. Several factors contribute to POF, including the presence of infected stones, preoperative bacteriuria, long operative times, and high intrarenal pressures during irrigation. Among these, preoperative urinary infection is the most preventable factor, highlighting the importance of reliable diagnostic tools for early detection of bacteriuria in surgical candidates [5,6].

Traditionally, urine culture has been considered the gold standard for diagnosing urinary infections and guiding perioperative antibiotic prophylaxis. Preliminary results, such as Gram stain or early growth reports, may be available within 24 hours; however, final identification and susceptibility results generally require up to 72 hours, which limits their practical value for immediate intraoperative decision-making [7].

In contrast, urine Gram stain (UGS) is a simple, rapid, and cost-effective diagnostic test that can detect the presence of bacteria within minutes [8]. Prior studies have reported UGS to have a sensitivity ranging from 70-80% and a specificity of approximately 85-90% for predicting urinary tract infection or postoperative infectious complications [3]. For example, renal pelvis UGS (RPUGS) in PCNL patients demonstrated 72.7% sensitivity, 89.9% specificity, and a strong independent association with postoperative fever (OR 15.0, 95% CI 5.4-41.2, p<0.001) [3]. By identifying bacteriuria early, UGS may enable timely antibiotic administration, reduce the risk of postoperative infectious complications, and guide intraoperative decision-making.

Despite its potential, UGS remains underutilised and underexplored in the context of urolithiasis and endourological surgery. Most existing literature has focused on its role in general urinary infections or pyelonephritis, with limited evidence regarding its predictive value for postoperative fever in patients undergoing stone surgery [9-11]. Given the rising burden of antimicrobial resistance, there is a pressing need for rapid, point-of-care diagnostic strategies that can support targeted prophylaxis and reduce unnecessary antibiotic use.

This prospective study aims to evaluate the predictive value of preoperative UGS for postoperative fever in patients undergoing endourological procedures for stone disease.

## Materials and methods

This prospective study was conducted at a tertiary care centre between January 2024 and July 2025 after obtaining approval from the Institutional Ethical Committee, BLDE (Deemed to be University), Vijayapura, with approval number BLDE(DU)/IEC/1065/2023-24. A total of 150 consecutive adult patients, aged 18 years or older, who underwent ureterorenoscopy (URS) or percutaneous nephrolithotomy (PCNL) or Retrograde Intrarenal Surgery (RIRS) for urolithiasis were enrolled after providing written informed consent. Patients with ongoing sepsis, recent antibiotic use within the preceding 14 days, positive preoperative urine culture, postoperative fever due to other identifiable causes (e.g., thrombophlebitis or chest infections), or incomplete clinical/microbiological data were excluded. The sample size was calculated using the formula, $n = \frac{Z^2 p q}{d^2}$, considering a postoperative fever prevalence of 15.6% as reported by Karsiyakali et al. [3], with a 95% confidence level and 6% allowable error, yielding a required sample of 141 patients, rounded to 150.

For all participants, preoperative midstream urine samples were collected under sterile precautions prior to surgery. These samples were subjected to both Gram staining and standard urine culture. Gram staining was performed and interpreted by a microbiologist who was blinded to clinical outcomes, and a urine Gram stain (UGS) was considered positive if ≥ 1 bacterium per high-power field was visualised under light microscopy. Urine culture was performed using conventional methods, and the result was considered positive if bacterial growth reached ≥105 colony-forming units (CFU) per millilitre. In addition to microbiological evaluation, demographic data, stone characteristics including size and location, and intraoperative details were systematically recorded. Postoperative fever (POF) was defined as a documented axillary or oral temperature ≥38 °C within 72 hours postoperatively, with follow-up extending to capture fever episodes occurring after discharge during this period.

All data were entered into a predesigned proforma and subsequently analysed using IBM SPSS Statistics for Windows, Version 27.0 (IBM Corp., Armonk, NY, USA). Categorical variables, such as UGS positivity and the presence of POF, were expressed as frequencies and percentages and compared using the chi-square test. Continuous variables, including stone size, were reported as mean ± standard deviation and compared using the independent-samples t-test. To determine independent predictors of POF, binary logistic regression analysis was performed. The diagnostic performance of UGS in predicting POF was assessed using receiver operating characteristic (ROC) curve analysis, with calculation of sensitivity, specificity, and area under the curve (AUC). A p-value of less than 0.05 was considered statistically significant for all analyses.

## Results

Out of a total of 150 patients who were included in the study, POF occurred in 24 patients (16.0%) within 72 hours of surgery, while 126 patients (84.0%) remained afebrile. None of the patients progressed to sepsis. The mean age was 47.1 ± 13.2 years, with no significant difference between those who developed POF (48.9 ± 12.8 years) and those who remained afebrile (46.7 ± 13.3 years, p=0.471). There were 84 male (56.0%) and 66 female (44.0%) patients, with a comparable gender distribution in both groups (p=0.694). URS was the predominant procedure, performed in 82 patients (54.6%), while PCNL was done in 46 patients (30.6%) and RIRS was done in 22 patients (14.6%). The mean stone size was 12.8 ± 5.9 mm, slightly larger in the POF group (14.1 ± 6.3 mm) than in the non-POF group (12.5 ± 5.8 mm, p=0.232) (Table 1).

**Table 1:** Baseline characteristics Categorical variables were expressed as frequencies and percentages and compared using the chi-square test. Continuous variables were reported as mean ± standard deviation and compared using the independent-samples t-test. POF: postoperative fever; URS: ureterorenoscopy; PCNL: percutaneous nephrolithotomy; RIRS: retrograde intrarenal surgery.

Variable		Total (n=150)	POF (n=24)	No POF (n=126)	Test statistic (χ² / t)	p-value
Age (years)	(mean ± SD)	47.1 ± 13.2	48.9 ± 12.8	46.7 ± 13.3	t = 0.724	0.471
Gender	Male	84 (56.0 %)	12 (50.0 %)	72 (57.1 %)	χ² = 0.155	0.694
	Female	66 (44.0 %)	12 (50.0 %)	54 (42.9 %)		
Procedure	URS	82 (54.6 %)	12 (50.0 %)	70 (55.6%)	χ² = 0.258	0.878
	PCNL	46 (30.6 %)	8 (33.3 %)	38 (30.2 %)		
	RIRS	22 (14.6%)	4 (16.7 %)	18 (14.3 %)		
Stone size (mm)	mean ± SD	12.8 ± 5.9	14.1 ± 6.3	12.5 ± 5.8	t = 1.198	0.232

Positive urine findings were strongly associated with the development of POF. Patients with positive UGS developed POF in 19 (79.2%) of cases compared to 21 (16.7%) with negative UGS (p<0.0001). Similarly, positive urine culture was observed in 16 (66.7%) of patients with POF versus 18 (14.3%) without POF (p<0.0001) (Table 2).

**Table 2:** Association of urine findings with POF Categorical variables were expressed as frequencies and percentages and compared using the chi-square test. POF: postoperative fever; UGS: urine Gram stain

Variable		Total (n=150)	POF (n=24)	No POF (n=126)	Test statistic (χ²)	p-value
UGS	Positive	40 (26.7 %)	19 (79.2 %)	21 (16.7 %)	40.26	<0.0001
	Negative	110 (73.3 %)	5 (20.8 %)	105 (83.3 %)		
Urine culture	Positive	34 (22.7 %)	16 (66.7 %)	18 (14.3 %)	31.55	<0.0001
	Negative	116 (77.3 %)	8 (33.3 %)	108 (85.7 %)		

**Figure 1:**
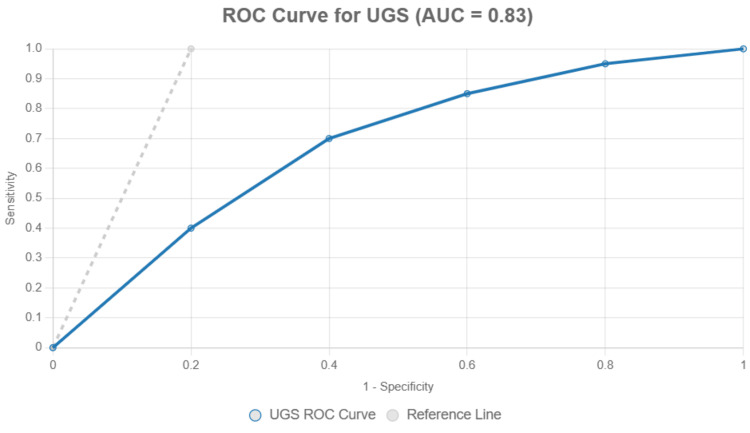
ROC curve for UGS in predicting POF The diagnostic performance of UGS in predicting POF was assessed using ROC curve analysis. UGS: urine Gram stain; POF: postoperative fever; ROC: receiver operating characteristic; AUC: area under the curve

Preoperative UGS exhibited 79.2% sensitivity, 82.5% specificity, 47.5% positive predictive value (PPV), and 94.5% negative predictive value (NPV) for predicting POF. The ROC curve analysis demonstrated an AUC of 0.83 (95% CI 0.74-0.92, p<0.001), reflecting strong predictive performance (Figure 1).

On multivariate analysis, positive UGS (OR 11.8, 95% CI 4.1-34.2, p<0.001) and positive urine culture (OR 5.9, 95% CI 2.1-16.7, p=0.001) were identified as independent predictors of POF, whereas age and sex were not significant (Table 3).

**Table 3:** Multivariate logistic regression for POF predictors To determine independent predictors of POF, binary logistic regression analysis was performed. UGS: urine Gram stain; POF: postoperative fever

Variable	OR (95% CI)	p-value
Positive UGS	11.8 (4.1–34.2)	<0.001
Positive urine culture	5.9 (2.1–16.7)	0.001
Age (per year increase)	1.01 (0.97–1.05)	0.589
Sex (Female vs. Male)	1.3 (0.5–3.6)	0.602

## Discussion

The emergence of POF following endourological interventions like URS, PCNL, and RIRS remains a pertinent clinical concern, with the potential to herald more severe infectious complications such as urosepsis. While preoperative urine culture (UC) is the gold standard for detecting bacteriuria, its delayed results limit its utility in immediate perioperative decision-making. This prospective study reinforces the clinical value of preoperative urine Gram stain (UGS) as a rapid and reliable tool to predict postoperative infectious events.

In the present study, POF occurred in 16% of patients undergoing endourological procedures, which is consistent with previously reported incidence rates ranging from 10% to 20% in similar cohorts [3,4]. Yang et al. reported the incidence of postoperative RIRS infection in 25.0% of patients [12]. The lack of significant association between age and the development of POF (p=0.471) suggests that postoperative infectious risk in endourological surgeries may be more strongly influenced by microbiological or procedural factors than by patient age alone. Endourological meta-analysis findings suggest that the type of procedure (RIRS vs. PCNL) does not significantly alter the odds of fever or sepsis, reinforcing that infection risk may lie elsewhere [13]. Retrospective analyses in broader urological surgeries also show that a positive preoperative urine culture, especially polymicrobial, elevates the risk of postoperative febrile infections (ORs ranging from ~2.8 to 3.7) even when controlling for other variables [14,15]. Similarly, an observational study on PCNL found that larger stones, staghorn calculus, prolonged operative time, significant bleeding, and positive preoperative urine culture were key predictors of postoperative sepsis, supporting the role of early infection detection tools like UGS [16].

While stone size was slightly larger in the POF group, the difference was not statistically significant (p=0.232), which mirrors findings by Gutierrez et al., who concluded that stone burden alone does not reliably predict infectious complications unless accompanied by bacterial colonisation or obstruction [10]. The distribution of procedures (URS 54.6%, PCNL 30.6%, RIRS 14.6%) reflects current urological practice patterns, and the lack of procedure-specific difference in POF incidence further emphasises the importance of preoperative infection screening over procedural type as a determinant of postoperative infectious risk.

The current study demonstrated a strong association between UGS positivity and POF, with a sensitivity of 79.2%, specificity of 82.5%, and AUC of 0.83. This is consistent with the findings of Karsiyakali et al., who reported that the sensitivity and specificity of RPUGS in predicting POF were 72.7% and 89.9% respectively [3]. They also found a positive correlation between Gram stain and stone culture, suggesting that UGS can indirectly reflect intrarenal microbial load. Yang et al. developed a nomogram for predicting urosepsis in RIRS patients with negative pre-op UC and highlighted the utility of integrating early diagnostic parameters for more effective perioperative planning [12].

The strong association between positive preoperative urine findings and POF in this study highlights the importance of early infection screening. A significantly higher proportion of patients who developed POF had a positive urine Gram stain (79.2%) and urine culture (66.7%) compared to those who remained afebrile. These findings are consistent with previous studies, including Karsiyakali et al., who reported a similar predictive value of renal pelvis Gram stain and culture in PCNL patients [3]. Yang et al. also demonstrated that occult bacteriuria, even in patients with negative cultures, could predispose to post-RIRS infections, reinforcing the value of rapid screening tools like UGS [12]. The statistically significant correlation (p<0.0001) observed in our study supports the use of UGS as a sensitive, fast, and practical marker for identifying patients at elevated risk of postoperative infectious complications. Similarly, in a UTI screening study by Wiwanitkit et al., UGS had a reported sensitivity of 96.2% and specificity of 93% in detecting bacteriuria, confirming its robust diagnostic value even outside the surgical context [17].

On multivariate analysis, both positive UGS and positive urine culture emerged as independent predictors of POF, with UGS showing a stronger association (OR 11.8) than urine culture (OR 5.9). These findings highlight the superior predictive power of UGS, likely due to its ability to rapidly detect bacteriuria prior to clinical manifestations. Similar trends were reported by Karsiyakali et al., who found Gram stain to be a stronger predictor of POF and stone culture positivity compared to traditional urine culture [3]. Additionally, Wang et al. and Yang et al. also identified preoperative bacteriuria as a key independent risk factor for infectious complications, even in patients undergoing minimally invasive procedures like RIRS [11,12]. The lack of association between demographic and procedural variables such as age, sex, stone size, and procedure type with POF in our study aligns with findings by Akgül et al., suggesting that microbiological factors outweigh anatomical or procedural characteristics in determining infectious risk [4]. This reinforces the clinical value of incorporating UGS into routine preoperative assessment for early risk stratification and antibiotic planning.

Strengths of this study include its prospective design and inclusion of both URS and PCNL cases, reflecting real-world practice. The systematic collection of demographic, procedural, and microbiological data, coupled with multivariate logistic regression, allowed us to affirm that UGS is an independent predictor of POF even after controlling for conventional factors such as stone size and procedure type.

However, certain limitations should be noted. This was a single-centre study with a relatively small sample size, which may limit the statistical power and affect generalisability to different settings or populations. Furthermore, only midstream urine samples were used; intraoperative renal pelvis urine or stone cultures were not collected, though such samples may more precisely reflect the true infective burden. Moreover, the study focused solely on POF as the outcome. While no cases progressed to sepsis, larger multicentre trials would be needed to confirm UGS’s ability to predict more severe outcomes like systemic inflammatory response or urosepsis. Additionally, potential confounding variables such as operative duration and prior ureteral stenting were not recorded and thus could not be included in the multivariate model. These factors may have influenced the observed association between preoperative UGS and POF and should be accounted for in future studies.

## Conclusions

Preoperative urine Gram stain is a rapid and cost-effective tool for predicting postoperative fever in patients undergoing endourological surgery for urolithiasis. Its ability to provide near-immediate results can help identify high-risk patients and guide timely antibiotic decisions, potentially reducing postoperative infectious complications.

## References

[1] Alghamdi AM, Alqurashi EA, Alsubaie AT (2025). Management of recurrent urolithiasis: advances in prevention strategies. Int J Community Med Public Health.

[2] Bhanot R, Jones P, Somani B (2021). Minimally invasive surgery for the treatment of ureteric stones - state-of-the-art review. Res Rep Urol.

[3] Karsiyakali N, Yucetas U, Karatas A, Karabay E, Okucu E, Erkan E (2021). Renal pelvis urine Gram stain as a traditional, but new marker in predicting postoperative fever and stone culture positivity in percutaneous nephrolithotomy: an observational, prospective, non-randomized cohort study. World J Urol.

[4] Akgül M, Özman O, Başataç C (2025). Can we predict postoperative fever and urinary tract ınfection after retrograde ıntrarenal surgery? Results of a case control matching multicentric RIRSearch study group. World J Urol.

[5] Peng L, Zeng Y, Wu Y, Yang J, Pei F, Shen B (2021). Preoperative bacteriuria positivity on urinalysis increases wound complications in primary total hip arthroplasty regardless of the urine culture result. BMC Musculoskelet Disord.

[6] Haskell-Mendoza AP, Radhakrishnan S, Nardin AL (2023). Utility of routine preoperative urinalysis in the prevention of surgical site infections. World Neurosurg.

[7] Xu R, Deebel N, Casals R, Dutta R, Mirzazadeh M (2021). A new gold rush: a review of current and developing diagnostic tools for urinary tract infections. Diagnostics (Basel).

[8] Davenport M, Mach KE, Shortliffe LM, Banaei N, Wang TH, Liao JC (2017). New and developing diagnostic technologies for urinary tract infections. Nat Rev Urol.

[9] Simon J, Kleinclauss F, Chabannes É, Bouiller K, Frontczak A (2024). Urinary tract infection after flexible ureterorenoscopy for urolithiasis in patients with positive treated preoperative urinalysis. Urolithiasis.

[10] Gutierrez J, Smith A, Geavlete P (2013). Urinary tract infections and post-operative fever in percutaneous nephrolithotomy. World J Urol.

[11] Wang L, Yu X, Qiu Z (2024). Influence of preoperative urine culture and bacterial species on urogenital sepsis after ureteral flexible lithotripsy in patients with upper urinary tract stones. Front Med (Lausanne).

[12] Yang M, Li Y, Huang F (2023). A nomogram for predicting postoperative urosepsis following retrograde intrarenal surgery in upper urinary calculi patients with negative preoperative urine culture. Sci Rep.

[13] Bapir R, Bhatti KH, Eliwa A (2022). Infectious complications of endourological treatment of kidney stones: a meta-analysis of randomized clinical trials. Arch Ital Urol Androl.

[14] Vallée M, Cattoir V, Malavaud S (2019). Perioperative infectious risk in urology: Management of preoperative polymicrobial urine culture. A systematic review. By the infectious disease Committee of the French Association of urology. Prog Urol.

[15] Kutchukian S, Gondran-Tellier B, Dinh A (2024). Asymptomatic bacteriuria and urological surgery: risk factor or not? Results from the National and Multicenter TOCUS Database. J Urol.

[16] Pratap V, Sayed AA, Kharade M (2024). An observational study of predictive factors for fever and sepsis following percutaneous nephrolithotomy. Int Surg J.

[17] Wiwanitkit V, Udomsantisuk N, Boonchalermvichian C (2005). Diagnostic value and cost utility analysis for urine Gram stain and urine microscopic examination as screening tests for urinary tract infection. Urol Res.

